# The roles of microglia in viral encephalitis: from sensome to therapeutic targeting

**DOI:** 10.1038/s41423-020-00620-5

**Published:** 2021-01-12

**Authors:** Chintan Chhatbar, Marco Prinz

**Affiliations:** 1 Institute of Neuropathology, Medical Faculty, University of Freiburg, Freiburg, Germany; 2 Center for Basics in NeuroModulation (NeuroModulBasics), Faculty of Medicine, University of Freiburg, Freiburg, Germany; 3 Signalling Research Centres BIOSS and CIBSS, University of Freiburg, Freiburg, Germany

**Keywords:** microglial activation, viral encephalitis, neurocognitive issues, purinergic signaling, PRR signaling, Neuroimmunology, Innate lymphoid cells

## Abstract

Viral encephalitis is a devastating disease with high mortality, and survivors often suffer from severe neurological complications. Microglia are innate immune cells of the central nervous system (CNS) parenchyma whose turnover is reliant on local proliferation. Microglia express a diverse range of proteins, which allows them to continuously sense the environment and quickly react to changes. Under inflammatory conditions such as CNS viral infection, microglia promote innate and adaptive immune responses to protect the host. However, during viral infection, a dysregulated microglia-T-cell interplay may result in altered phagocytosis of neuronal synapses by microglia that causes neurocognitive impairment. In this review, we summarize the current knowledge on the role of microglia in viral encephalitis, propose questions to be answered in the future and suggest possible therapeutic targets.

## Introduction

The central nervous system (CNS) consists of cells derived from two distinct developmental lineages, neuroepithelial progenitors, and erythromyeloid precursor (EMP) cells. Neurons, astrocytes, and oligodendrocytes arise from self-renewing pluripotent neuroepithelial progenitors.^1^ Microglia are long-lived innate immune cells of the CNS parenchyma that arise from EMP cells and populate the neuroectoderm early in development.^2–5^ All these cell types (together with epithelial cells, endothelial cells, and pericytes) are essential for the formation of the unique physiological environment required for the optimal functioning of the CNS.

Although the CNS is separated from the periphery by several different types of barriers, pathogens have evolved ways to reach the CNS via different routes. Upon entry of a pathogen into the CNS, a wide range of consequences are possible, depending on the host and the pathogen. For example, herpesviruses remain latent in an immunocompetent host for life; however, they can also cause acute herpes encephalitis when the immune system is unable to control the virus, e.g., under conditions of immune senescence in advanced age or in the presence of primary and acquired immunodeficiency. At the other extreme, rabies virus (RABV) causes an acute CNS infection that is always fatal.^6^ Frequently, the direct and indirect neuronal damage due to acute infection or reactivation of a virus within the CNS and the immune response in the CNS leads to long-lasting neurobehavioral issues in recovered patients. High susceptibility of the CNS to pathogens and immune-mediated damage together with a lack of regenerative ability result in permanent damage and reduced CNS functionality.^7^ Other factors that result in high morbidity and mortality due to CNS infections are the incomplete knowledge of specific disease pathogeneses, lack of tools for the diagnosis and insufficient options for treatment.^6,8,9^ Until recently, the exact mechanisms of CNS destruction were not known, and recent studies have shown temporal and spatial roles for microglia in CNS protection and destruction. As human contacts with vectors of zoonotic pathogens cannot be eliminated, our understanding of the pathogeneses of zoonotic diseases must be improved, especially with regard to possible targets for treatment. In this review, we present models to show the potential roles of microglia in viral encephalitis on the basis of current knowledge and suggest that microglia-targeting therapeutics can be used for the treatment of viral encephalitis-associated neurocognitive complications.

## Viral encephalitis has high morbidity and leads to long-lasting neurocognitive sequelae

Several viruses cause encephalitis. RNA viruses that cause encephalitis in humans are typical zoonotic infections. Virus “jumping” from its natural host, e.g., birds or animals, into humans results in zoonotic infections.^10^ Important RNA viruses that cause encephalitis in humans include those of the Flaviviridae family, Togaviridae family, and Rhabdoviridae family.^6^ Rabies virus, which belongs to the Rhabdoviridae family, is a classic example of an acute zoonotic infection that causes 60,000 deaths worldwide every year.^6,11^ Japanese encephalitis virus (JEV), Dengue virus (DenV), West Nile virus (WNV), and Zika virus (ZIKV) belong to the Flaviviridae family. JEV is currently restricted to Southeast Asia, where it causes as many as 50,000 infections with a fatality rate as high as 30%. Among survivors of JEV encephalitis infection, 30–50% have significant neurological, cognitive, or psychiatric sequelae.^12,13^ DenV has global prevalence. Estimates suggest that ~390 million DenV infections occur annually, and in some cases, as many as 21% of these patients present with neurological involvement.^6,14^ WNV is responsible for encephalitis-associated morbidity, mortality, and postrecovery neurocognitive deficits in America.^7^ Additionally, the newly emerging ZIKV may cause similar or different long-term sequelae in survivors. Alphaviruses are enveloped, single-stranded positive-sense RNA viruses of the Togaviridae family that are transmitted by infected biting mosquitos, making them arthropod-borne viruses.^15^ Venezuelan, Western, and Eastern equine encephalitis viruses (VEEV, WEEV, and EEEV, respectively) are major encephalitic alphaviruses that involve high morbidity and neurological sequelae.^15^ Highly adapted human viruses, such as herpesviruses, can cause chronic CNS infections, which can persist throughout the life of immunocompetent individuals without causing severe CNS conditions. Among herpesviruses, herpes simplex virus (HSV) infections occur worldwide and can cause HSV encephalitis, which is fatal without treatment.^6,10,16^ Congenital cytomegalovirus infections in newborns also lead to developmental delay in 50% of the affected children.^6^

It is well documented that CNS inflammation or altered functioning of CNS cells leads to a wide range of behavioral changes.^17^ In the case of viral encephalitis, viral infection and immune response-mediated changes in gene expression in combination bring multiple changes to CNS physiology, which ultimately affects short and long-term behavior. Although acute behavioral symptoms are lost after resolution of the infection, ongoing immune responses, even after pathogen clearance, cause long-term psychiatric, neurocognitive, and degenerative issues in survivors.^18^ Neurocognitive complications of viral encephalitis include memory disorders, cognitive deficits such as learning disabilities, motor deficits, and changes in mood and personality. A total of 30–50% of people recovering from viral encephalitis develop one or more of these symptoms. Another common result of viral encephalitis is the development of seizures. All of these factors lead to difficulties in daily life.^6,18,19^

In a study providing prospectively acquired neurological outcomes, the data among American patients with WNV-induced CNS disease showed that during initial clinical presentation, 93% of the patients exhibited a significant neurological deficit, and almost one-half of them had cognitive deficits after a 90-day follow-up.^20^ After recovery from the disease, the patients who exhibited neurological deficits 90 days after their initial evaluation constituted only 20% of the study cohort.^20^ In a long-term observational study of neurological abnormalities 1–3 and 8–11 years following WNV infection, 86% of patients with WNV encephalitis had abnormal neurological findings at the time of the first assessment, but not uncomplicated fever or meningitis.^21^ Interestingly, at the time of the second assessment, ~40% of all patients evaluated had developed new neurological complications.^21^ This study showed that new neurological complications can develop long after viral clearance. The most common physical, cognitive, and functional sequelae associated with WNV encephalitis are muscle weakness, memory loss, and difficulties in daily living. Other problems include hearing loss and abnormal reflexes. Long-term neurological abnormalities occur most commonly in patients who suffer from WNV encephalitis. Thus, a clinical presentation of West Nile encephalitis (WNE) is associated with serious neurological complications.^21,22^

In addition to WNV, other neuroviral pathogens associated with neurocognitive impairment include DENV, JEV, Nipah virus, and alphaviruses. Neurological complications associated with DENV encephalitis include neuromuscular complications, such as Guillain-Barre syndrome, rhabdomyolysis, transient muscle dysfunctions, and neuro-ophthalmic involvement.^6,14^ Neurological complications associated with alphavirus encephalitis include confusion, visual disturbances, photophobia, seizures, somnolence, coma, intellectual disability, and emotional instability/behavioral changes.^15^ Surprisingly, 50–90% of survivors of Eastern equine virus (EEV) encephalitis showed these neurological sequelae.^15^ Nipah virus encephalitis also seems to be associated with more neuropsychiatric sequelae than neurocognitive sequelae. It has been reported that recovered patients suffer from major depressive disorder, personality changes, chronic fatigue syndrome, and substantial deficits in attention and verbal and/or visual memory.^23^ Neurological manifestations and outcomes are also reported for patients infected with the 2009 H1N1 virus infection, especially children. The most common neurological presentations of the H1N1 virus include seizures and acute necrotizing encephalopathy (ANE) requiring hospital admission and resulting in high mortality and morbidity.^24,25^ Thus, the neurological complications caused by viral encephalitis are highly variable depending on the infectious agent. One possible explanation involves bystander effects of the host response towards the pathogen.

## Distinct roles of microglia and monocytes in viral encephalitis

**Fig. 1:**
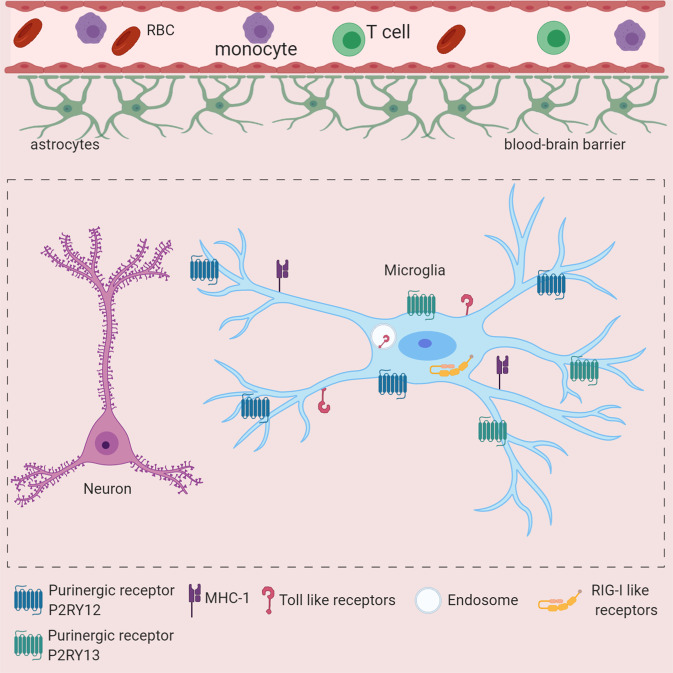
The microglial sensome is critical for the response to CNS viral infection. Schematic depiction of important microglial sensors involved in virus infection-mediated damage sensing. Toll-like receptors (TLRs) are located on the cell surface or in endosomal compartments. TLR3 and TLR7 are involved viral genome sensing. RIG-I-like receptors such as RIG-I, MDA5, and cGAS are localized to the cytoplasm and sense the viral genome in the cytoplasm. The purinergic receptor P2RY12 is involved in ATP sensing and regulates microglial recruitment after cell damage. P2RY13 may play a role in microglial activation and responses upon ADP sensing during inflammation. Microglia express MHC-II molecules and may cross-present antigens to regulate T-cell responses in the CNS

Different long-lived tissue-specific myeloid cells, such as microglia and dural, leptomeningeal, perivascular, and choroid plexus macrophages, collectively known as CNS-associated macrophages (CAMs), populate the healthy CNS.^26^ Among these, microglia reside in the CNS parenchyma and expresses the myeloid cell marker CX3CR1. In contrast to other tissue-specific myeloid cells, microglia, and most other CNS-specific myeloid cells derive from c-Kit^lo^CD41^lo^ progenitors present in the yolk sac (YS) and are not derived through definitive hematopoiesis.^2–5^ Due to the presence of the blood-brain barrier (BBB), which physically isolates the CNS from the blood circulation in adulthood, entry of blood-derived myeloid cells under homeostatic conditions does not occur; thus, the turnover of microglia depends on local homeostatic proliferation.^27^ Previously, a multicolor fluorescence fate-mapping system revealed microglial turnover under homeostatic conditions. Interestingly, depending on the brain region, microglia show different turnover rates. Olfactory bulb (OB) microglia have high turnover rates, whereas microglia from the cortex have very low turnover rates.^28^ Thus, embryonic origin, self-renewal without contributions from definitive hematopoiesis and the specific CNS environment, such as interaction with neurons, oligodendrocytes, etc., make microglia a unique tissue-residing myeloid cell population. In the resting state, microglia use processes and protrusions, endowing these cells with high motility, to continuously survey the CNS microenvironment.^29,30^ The high sensitivity of microglia to changes in the CNS microenvironment is due to the expression of a large repertoire of proteins that allow them to sense invading pathogens, dying cells, and exogenous and endogenous ligands, which is defined as the “microglial sensome”^31^ (Fig. 1).

CNS viral infections lead to the accumulation of myeloid cells in different CNS regions.^32–36^ Ionized calcium-binding adapter molecule-1 (Iba-1) is a commonly used marker to investigate the myeloid cell response in the CNS upon viral encephalitis.^8,37^ However, Iba-1 is also expressed by monocytes.^38^ During neuroinflammation, it impossible to distinguish microglia and monocytes via such common markers.^38^ Limited by the prevalent knowledge of the time, previous studies proposed that circulating monocytes infiltrate the brain in a CCR2-dependent manner and gave rise to microglia during viral encephalitis,^39^ where CNS inflammation leads to preconditioning of the CNS that allows the migration of monocytes into the brain.^40^ Depending on the model, both pathogenic and protective roles were described for these monocyte-derived “microglia”.^39,41^ In contrast to YS-derived microglia, monocytes are generated in the bone marrow during definitive hematopoiesis, are short living and are of two types: Ly6C^hi^ monocytes, the so-called inflammatory tumor necrosis factor (TNF)-α^+^ and CD11c^+^ monocytes, and Ly6C^lo^ monocytes, which have patrolling functions in blood vessels.^42^ We have previously used a genetic labeling approach with tamoxifen-inducible Cx3cr1cre^ER/+^:R26Rtomato^St/Wt^ reporter mice to distinguish microglia and monocytes during viral encephalitis.^43,44^ Using a nasally infected (i.n.) vesicular stomatitis virus (VSV) infection model^32,45,46^ and Theiler’s murine encephalomyelitis virus (TMEV) model of encephalitis-associated seizures and neurodegeneration,^19,33^ we showed that microglia accumulate by local proliferation in the CNS upon viral infection.^32,33^ These studies also showed that upon viral encephalitis, infiltrating Ly6C^hi^ monocytes do not give rise to microglia.^32^ We have shown the infiltration of the CNS by monocytes in VSV encephalitis and TMEV encephalitis-associated seizures.^32,33^ Similarly, others have shown the infiltration of the CNS by monocytes in WNV encephalitis^39,41^ and in a murine model of encephalitis by CD8^+^ T cells targeting viral antigen-positive neurons.^47^

The critical roles of microglia in viral control within the CNS and host protection have been debated for a long time. Clear experimental evidence for a protective role by microglia was lacking because methods to deplete microglia with minimal perturbation of the peripheral immune response were missing. With the development of pharmacological agents that inhibit colony-stimulating factor 1 receptor (CSF-1R) signaling and thus allow the depletion of CSF-1R-signaling-dependent cells, i.e., microglia in the CNS, several studies have proposed that microglia might be critical for host protection during viral encephalitis.^32,35,48–50^ These studies have examined different routes of CNS infection, such as intracranial and intranasal, from the peripheral nervous system and peripheral infection that caused encephalitis after viral entry into the CNS. The protective role of microglia has been shown in viral encephalitis induced with diverse flaviviruses, such as WNV and JEV,^50,51^ mouse hepatitis virus (MHV) which is a neurotropic coronavirus,^35^ an i.n. administered VSV infection model,^32^ TMEV model of encephalitis-associated seizures and neurodegeneration,^34^ and pseudorabies virus (PRV) model of herpesvirus encephalitis.^52^ Together, these studies suggested that microglia are essential for host protection during viral encephalitis.

Chemokine receptor 2 (CCR2) is essential for Ly6C^hi^ monocyte accumulation in the CNS after viral infection and for the induction of autoimmune encephalomyelitis.^33,39,41,47,53^ Ly6C^hi^ monocyte accumulation in the CNS upon viral encephalitis seems to be important for host protection, as CCR2^−/−^ mice showed enhanced susceptibility to WNV encephalitis.^41^ However, depletion of peripheral blood monocytes by clodronate treatment or inhibition of monocyte entry in the CNS by anti-C-C motif chemokine ligand (CCL2) antibody treatment in a model of lethal WNV encephalitis led to enhanced protection, implying a pathological role of Ly6C^hi^ monocytes in WNV encephalitis.^39^ Similarly, using Theiler’s virus model, we also showed that macrophage depletion via chlodronate liposome treatment of animals suppressed seizure development,^54^ and CCR2^−/−^ mice showed a limited death of hippocampal neurons,^33^ pointing towards a pathological role for infiltrating Ly6C^hi^ monocytes in viral encephalitis neurodegeneration. Monocyte-derived macrophages also take part in synaptic stripping, similar to microglia; however, this process seems to be undertaken independent of complement pathway activation.^47^ In conclusion, Ly6C^hi^ monocytes have a complex role in viral encephalitis.

## Roles of purinergic receptors during viral encephalitis

Purinergic receptors constitute an evolutionarily conserved family of receptors that bind to nucleotides and regulate cellular responses in an autocrine and paracrine manner. There are three families of purinergic receptors, and depending on the immune response elicited, they are classified into two types.^55^ P1 receptors include four G protein-coupled adenosine receptors that induce an anti-inflammatory response upon binding to adenosine. P2 receptors include six P2X purinergic receptor (P2XR) homotrimers, four P2XR heterotrimers and eight P2YR G protein-coupled receptors (GPCRs) that bind to adenosine triphosphate (ATP) and/or other purine or pyrimidine nucleotides and induce a proinflammatory response.^55^ Microglia express several P1 and P2 purinergic receptors.^56^ Under homeostatic conditions, purinergic receptors are key regulators of microglial function, including but not limited to microglial motility and surveillance of the CNS, regulating neuronal activity and functions, the production of cytokines, and even the regulation of adult hippocampal neurogenesis.^56–60^ However, the proviral and antiviral roles of purinergic receptors in viral encephalitis and associated complications need further investigation.

Experiments of human macrophage infection with HIV in vitro have shown that P2 purinergic receptors are necessary for HIV infection. Pharmacological inhibition of purinergic receptors during macrophage exposure to HIV showed that P2X1, but not P2X7 or P2Y1, is necessary for HIV entry into macrophages and that P2X1, P2X7, and P2Y1 receptors are involved in HIV replication. Mechanistically, HIV binding to macrophages triggers a local release of ATP that stimulates purinergic receptors and facilitates HIV entry and the subsequent stages of viral replication.^61,62^ In addition to viral entry and replication, the role of purinergic receptors is also described in HIV-associated sensory neuropathy, a condition that includes pain, burning, and numbness due to the damage of nerve fibers that innervate distal limbs, particularly the feet.^63,64^

**Fig. 2:**
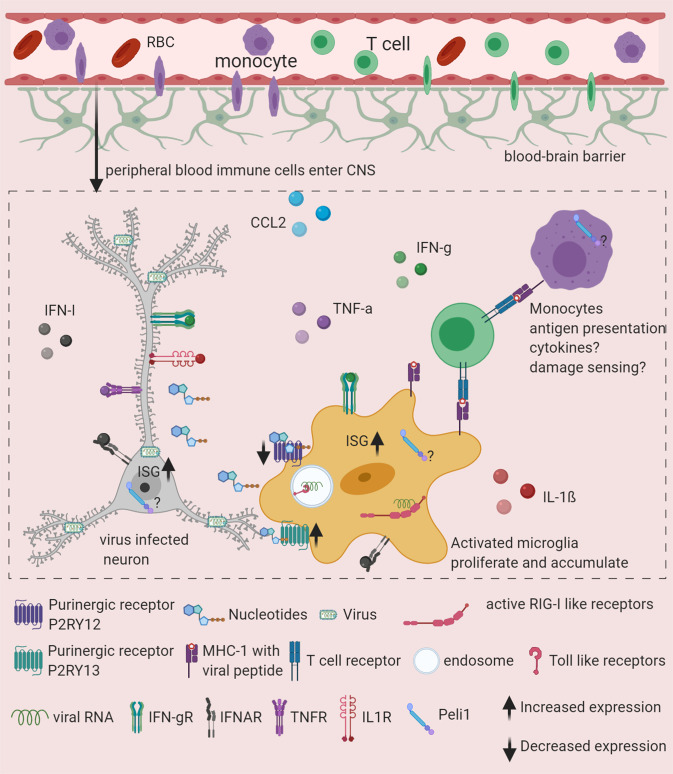
Microglia rapidly respond to CNS viral infection and play critical roles in orchestrating innate and adaptive responses to protect the host. Upon infection and damage, cells secrete nucleotides that microglia sense via purinergic receptors, inducing their subsequent migration to damaged cells. The sensing of damage-associated molecular patterns (DAMPs) leads to microglial activation, which enhances their capacity to sense viral pathogen-associated molecular patterns (PAMPs) and resulting cell damage. Interacting with infected cells allows microglia to sense viral PAMPs and acquire antigens for presentation to T cells. PAMP sensing leads to cytokine responses by microglia. Peli1, which regulates the NF-kB pathway, seems to be critical in inflammatory responses after CNS viral infection. Antigen presentation by microglia is thought to be critical for T-cell responses in the CNS and host protection. Very few studies have been able to assess the cross talk between components of these important functions of microglia. Furthermore, whether one microglial cell is able to perform diverse functions or whether labor is divided among cells in this context remains unknown.

In the pseudorabies virus (PRV) infection of the CNS model, which is a mouse model for herpesvirus infection, it has been shown that ATP released from virus-infected neurons leads to the rapid recruitment of microglia, which is mediated via ATP sensing by the P2RY12 receptor on microglia.^52^ Microglial recruitment to infected neurons leads to the phagocytosis of debris from infected neurons, which seems to be critical for inhibiting virus spread in the CNS.^52^ These data are supported by the presence of P2RY12-expressing microglia near virus-infected neurons in postmortem samples.^52^ In vitro experiments with microglia lacking P2RY12 showed that adenosine diphosphate (ADP) binding to P2RY12 augments inflammasome and nuclear factor- κB (NF-κB) activation, which enhances interleukin-1 (IL-1)β release from microglia.^65^ Given that IL-1β has been shown to have antiviral activity during WNV-induced encephalitis,^66^ P2RY12 signaling-mediated enhancement of IL-1β expression can potentially contribute to the restriction of viral spread and lethality. In vitro studies with bone marrow-derived macrophages showed that P2RY13, but not P2RY12, may be an interferon-stimulated gene (ISG), as P2RY13 expression is enhanced after TLR3 stimulation or interferon (IFN) treatment.^67^ Interestingly, it has been shown that P2RY12 expression is decreased in microglia during the development of viral encephalitis^32^ and other pathologies.^68^ Since microglia express both P2RY12 and P2RY13 under homeostatic conditions,^56^ we propose a model in which P2RY12 signaling on microglia allows their recruitment to infected/injured cells during the early events after viral infection of CNS cells. However, at later stages, P2RY13, whose expression is maintained or even increased after IFN stimulation of microglia, senses nucleotides and regulates microglial gene expression (Fig. 2).

Since there are many pharmacological agents available that target purinergic receptors in a specific manner, in vivo studies aimed at understanding the impact of purinergic receptor signaling in viral encephalitis have the potential to deliver  treatments for better outcomes of encephalitis.

## Microglial pathogen sensing and the immune response upon viral infection

Pattern recognition receptors (PRRs) are diverse germline-encoded families of membrane bound or cytoplasmic proteins that have evolved to recognize components of foreign pathogens referred to as pathogen-associated molecular patterns (PAMPs). In addition, these PRRs can also recognize endogenous molecules released from damaged cells, which are called damage-associated molecular patterns (DAMPs). Two major families of PRRs that engage in viral PAMP recognition are Toll-like receptors (TLRs) and retinoic acid inducible gene (RIG)-I-like receptors (RLRs). Sensing viral PAMP by PRR induces signaling cascades, which ultimately result in the expression of antiviral interferons and other proinflammatory cytokines.^69–71^ Several TLRs, RLRs, and other miscellaneous PRRs, such as the NLR family pyrin domain-containing 1 (NLRP1) inflammasome and DNA-dependent activator of IFN-regulatory factors (DAI), detect viral PAMPs.^72,73^ Microglia, as innate immune cells of the CNS parenchyma, express all TLRs, which can recognize viral genomes in the environment and endosomal compartments. Microglia also express retinoic acid inducible gene I (RIG-I) and melanoma differentiation-associated protein 5 (MDA5), which recognize viral genomes in the cytoplasm, and the cyclic GMP-AMP synthase (cGAS)-stimulator of interferon genes (STING) pathway, which is involved in the recognition of cytoplasmic DNA^37,74,75^ (Fig. 1).

Because of the inability to distinguish between myeloid cells in infected CNS without genetic labeling,^32^ very few studies have been able to show the exact role of cytokine responses of microglia in viral encephalitis. In vivo studies of different viral encephalitis models and in vitro exposure of primary microglia and mouse and human microglial cell lines to TLR3 ligand poly(I:C) or TMEV and cGAS ligand cyclic guanosine monophosphate–adenosine monophosphate (cGAMP) or HSV-1 showed the expression of several cytokines and chemokines including IFN-β, IFN-γ, TNF-α, IL-1β, CCL2, CCL5, and IL-6.^37,76–81^ These proinflammatory molecules induce neuronal death by causing direct and indirect neurotoxicity. TNF-α signaling in microglia upon JEV infection leads to glutamate release, contributing to neuronal death.^82^ Other inflammatory mediators, such as nitric oxide (NO), produced by microglia can lead to direct neuronal death^78^ (Fig. 2).

Recently, the Peli1 protein, which is an E3 ubiquitin ligase, has been implicated in promoting viral replication and inflammation in the CNS in mouse models of VSV and WNV encephalitis and in a mouse model of congenital Zika syndrome.^80,83,84^ In a mouse model of congenital Zika syndrome, Peli1 promotes inflammation by mediating NF-κB activation and the expression of IL-1β, TNF-α, and IL-6. Blocking Peli1 attenuated congenital malformations in mice,^84^ confirming a proinflammatory role in this model. Mice that do not express Peli1 showed reduced viral titers in the CNS and had increased survival upon a lethal VSV or WNV encephalitis challenge compared to WT mice. In the VSV encephalitis model, at least, Peli1 was found to be a negative regulator of type I IFN expression within the CNS. Since type I IFN expression and signaling in the CNS are critical for host protection after viral encephalitis,^45,46^ the authors proposed that Peli1-KO mice have better survival than WT mice. It has been established that Peli1 mediates chemokine and proinflammatory cytokine production, and Peli1-KO mice have decreased proinflammatory cytokine responses in the CNS in viral encephalitis and other CNS pathologies, such as autoimmune encephalomyelitis, which is a mouse model of multiple sclerosis.^80,83,85^ Thus, the higher susceptibility of WT mice may be a result of an exacerbated proinflammatory response in the CNS, not a lack of IFN response. Therefore, it has been proposed that Peli1 maintains the balance between the proinflammatory response and the type 1 IFN response in inflammation.

Studies to understand the role of Peli1 were performed with mice that lack Peli1 gene expression in all cell types in the body, and the response of individual cells to cytokine responses was not determined in vivo. Thus, the observations made in viral encephalitis models may not be specific for microglia, and cytokine responses may be induced by other types of cells. Neurons are major targets of both VSV and WNV.^36,86^ Neurons actively produce cytokines and regulate peripheral immune cell recruitment in the CNS upon viral infection.^47^ In fact, Peli1 was also highly expressed on WNV-infected neurons and adjacent inflammatory cells in postmortem brains from patients who died of acute WNV encephalitis.^80^ Regardless of the precise role of Peli1 in microglia, targeting Peli1 may regulate CNS inflammation during viral encephalitis, as Peli1 regulates proinflammatory responses in several inflammatory conditions.

In conclusion, microglia mount cytokine responses during viral encephalitis; however, the mechanisms by which microglial cytokine responses are induced and their impact on the outcome of viral encephalitis and associated neurological complications are not completely known due to a lack of studies that clearly distinguish microglial contributions in vivo.

## Microglia as nonredundant antigen-presenting cells during viral infection

During viral encephalitis, T-cell infiltration of the CNS is essential for host protection.^86–89^ Studies have shown that major histocompatibility complex (MHC) class I and II proteins and costimulatory molecules CD40 and CD86 are expressed on the surface of activated microglia following viral infection, and thus, the peptides in the MHC class I and MHC class II complexes are presented to CD8^+^ and CD4^+^ T cells, respectively.^86,90,91^ However, whether microglia are essential antigen-presenting cells in the CNS for host protection is not clear from the evidence obtained thus far. The depletion of microglia during encephalitis highlights their role as nonredundant antigen-presenting cells within the CNS. Microglial depletion via broad nonspecific pharmacological treatment with PLX5622 during mouse hepatitis virus (MHV) encephalitis led to higher infiltration of T cells in the CNS but decreased IFN-γ responses of the CD4^+^ T cells in the CNS.^35^ In-line with these findings, another study showed that during WNV encephalitis, microglia presented the viral antigen and costimulatory molecules to T cells that entered the CNS and thus facilitated the reactivation of T cells in the CNS.^51^ This reactivation of T cells seemed to be essential for host protection from lethal encephalitis.^51^ We have reported similar findings in which microglia-modulated T-cell activation during TMEV encephalitis and microglial depletion by PLX5622 accelerated the frequency of seizures and exacerbated hippocampal damage and neurodegeneration.^34^ Cross-presentation is the ability of antigen-presenting cells (APCs) to take up, process and present extracellular antigens on MHC class I molecules, that are normally presented on MHC class II, to activate cytotoxic CD8^+^ T cells.^92^ Due to cross-presentation, APCs can take up exogenous antigens from infected non-immune cells, e.g., neurons, and present them to cytotoxic T cells. This is critical for the initiation of cytotoxic T lymphocyte (CTL) responses to viruses that infect nonhematopoietic cells.^93^ Dendritic cells are best known for cross-presentation, but adult microglia are also able to cross-present antigens. Microglia purified from adult mice injected intracerebrally with OVA efficiently stimulated OVA-specific CD8^+^ T cells, suggesting that microglia can take up exogenous antigens and cross-present them on MHC-I molecules in vivo.^94^ In the VSV encephalitis model, microglia internalize antigens from infected neurons and cross-present them to antiviral CD8^+^ T cells, which allows the noncytolytic clearance of infection from neurons^86^ (Fig. 2). In conclusion, microglia not only play important roles early in CNS viral infection but also continue to be important during the chronic phase to promote adaptive immune responses and protect the host.

## Microglia cause CNS damage during the chronic phase of viral encephalitis, leading to neurodegeneration and neurocognitive impairments

**Fig. 3:**
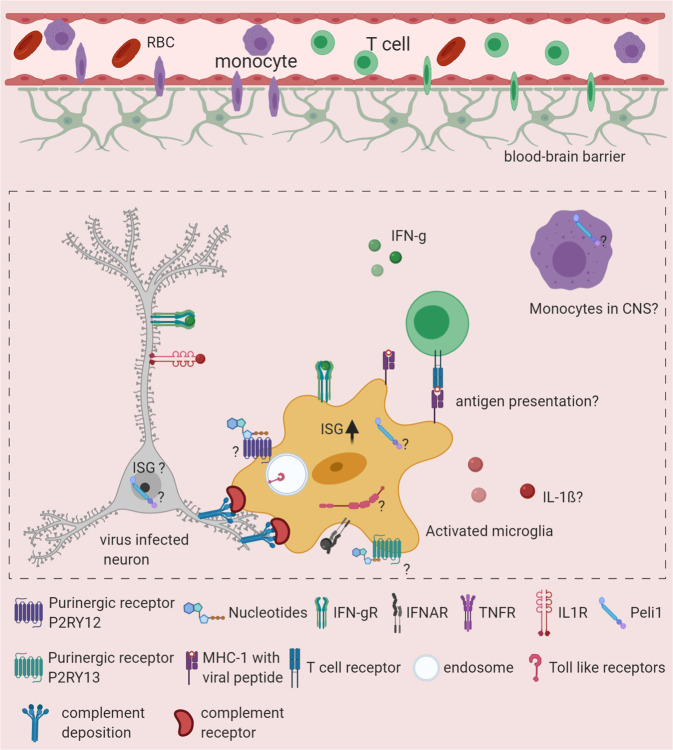
Microglia damage to neurons after encephalitis leads to neurological complications in survivors. Activated microglia are highly phagocytic and express many components of the complement pathway. Aggregation of complement components on neurons is sensed by complement receptor 3 (CR3), which is expressed by microglia. This activation leads to phagocytosis and synaptic stripping of neurons. Interferon gamma receptor (IFNgR) signaling in microglia regulates neuronal synaptic stripping upon the induction of viral encephalitis. The role of purinergic receptors, Peli1 and other cytokines in synaptic stripping is not known

The mechanisms underlying the neurocognitive issues related to viral encephalitis are not completely understood. However, recent studies have shed some light on the molecular mechanisms involved in this process. The classical complement cascade is a key component of the innate immune system and is involved in the direct killing of pathogens. Many components of the complement pathway are also expressed by microglia and other CNS cells, as this pathway is involved in synaptic pruning by microglia during early postnatal development.^95^ Experiments of infection with mutant strains of WNV and ZIKV that allowed greater animal survival after encephalitis showed that mice that recovered from acute encephalitis exhibited poor spatial-learning capacity.^8,36^ In WNV-infected animals, mice with poor spatial learning showed increased expression of genes that drive synaptic stripping by microglia via the complement pathway.^8^ In-line with this finding, WNV infection of adult hippocampal neurons leads to complement-mediated elimination of presynaptic terminals without neuronal loss.^8^ These observations made in a mouse model of viral encephalitis were corroborated with studies of patient samples. A postmortem study of human WNV encephalitis specimens showed loss of hippocampal CA3 presynaptic terminals.^8^ Evidence for the direct involvement of microglia and the complement cascade in CNS damage comes from experiments in which the infection of Il34(−/−) mice with decreased numbers of microglia or C3(−/−) and C3ar1(−/−) mice with complement C3 or C3a receptor deficiency were protected from WNV-induced synaptic terminal loss.^8^ In contrast to the synaptic stripping of neurons observed upon WNV encephalitis, ZIKV infection of the CNS in mice led to extensive neuronal apoptosis and loss of postsynaptic termini.^36^ These studies showed the mechanistic role of microglia in pathological synaptic stripping and neuronal apoptosis leading to memory impairment upon viral encephalitis (Fig. 3).

Synaptic stripping and neuronal apoptosis by myeloid cells seems to be the common mechanism of CNS damage upon the induction of the antiviral immune response, as this also occurs in the context of CD8^+^ T-cell-driven neuronal damage in a viral déjà vu model.^47^ In this model, upon re-exposure to the virus in the periphery, LCMV carrier animals expressing viral antigen in the CNS after viral elimination mount CD8^+^ T-cell-mediated immune responses in the CNS.^47,96^ Although myeloid cells are directly responsible for neuronal damage, the cross talk between CD8^+^ T cells, infected neurons, and myeloid cells drives the process of neuronal damage, as animals deficient in CD8^+^ T cells demonstrate protection against neuronal damage after viral encephalitis.^36,47^

Since CD8^+^ T cells mount IFN gamma (Ifn-γ) responses in the CNS during encephalitis, the role of IFN gamma (Ifn-γ) receptor (IfngR) signaling in neuronal damage has been investigated using Cx3cr1^creER/+^:Ifngr^fl/fl^ mice.^36^ In these mice, tamoxifen injection after viral elimination from the CNS allowed the deletion of Ifn-γ receptor (IfngR) signaling in CNS resident CX3CR1^+^ myeloid cells, including microglia. WNV and ZIKV infection of these mice followed by tamoxifen injection showed that, upon sensing of T-cell-derived Ifn-γ, CX3CR1^+^ cells such microglia potentiated spatial-learning defects after viral clearance.^36^ In the viral déjà vu model, T-cell-derived Ifn-γ also played a pathological role, but in contrast to direct stimulation of microglia, in the viral déjà vu model, Ifn-γ stimulation of neurons led to Ccl2 production in the neurons, which mediated phagocyte recruitment to these neurons.^47^

In conclusion, upon stimulation by T-cell-derived cytokines such as Ifn-γ, microglia mediate CNS damage by different mechanisms, such as synaptic stripping and inducing neuronal apoptosis in the virally infected CNS.

## Future perspectives

Microglia are innate immune cells residing in the CNS parenchyma and have a “sensome” that identifies and isolates the virus-infected neurons by surrounding them. Local proliferation of microglia during encephalitis allows the formation of this innate immune barrier, which prevents virus spread in the CNS soon after infection. During the chronic phase, microglia are possibly essential APCs that restimulate T cells in the CNS to promote adaptive immunity, which is essential for host protection. However, microglia can also damage neurons in this process, which leads to viral encephalitis-associated neurocognitive alterations. While studies have shown key receptors involved in these processes, the mechanistic details are still unclear. Purinergic receptors regulate microglial motility, but the dynamics of different purinergic receptors in microglia during viral encephalitis and their impact on microglial responses in vivo are not known.^56^ Very few studies have shown the role of microglia in virus sensing via PRRs in vivo. Type I IFN receptor (IFNAR) signaling-mediated regulation of microglia by other CNS cells is essential for microglial activation and host protection, but how IFNAR signaling mechanistically regulates microglial activation and the microglial sensome are not well known.^32^ Understanding these mechanisms will allow the development of novel treatment options for acute viral encephalitis. The interaction of T cells with microglia during viral encephalitis seems to be essential for host protection^86^ but also leads to microglia-mediated synaptic stripping. In the models of WNV and ZIKV encephalitis recovery in adult animals, pre- and postsynaptic neurons were damaged, respectively, which led to cognitive dysfunction. Interestingly, in both models, IFNgR signaling in microglia was critical for synaptic stripping.^36^ Di Liberto et al. showed that STAT1 signaling in neurons regulates microglia/macrophage-mediated synaptic stripping.^47^ Thus, IFNgR signaling seems to be critical in neuronal damage. However, whether IFNgR signaling is a common underlying mechanism for different neurocognitive issues after viral encephalitis remains to be investigated. Nevertheless, gaining a detailed understanding of IFN-γ mediated communication between different cell types after viral encephalitis may reveal clues for developing targeted therapies that inhibit synaptic stripping. Since the complement pathway is critical to synaptic stripping, the complement pathway is an obvious target. Similarly, Peli1 seems to be one of the key regulators of microglia-mediated CNS inflammation after viral encephalitis, which makes Peli1 a possible therapeutic target. It was shown in a model of multiple sclerosis that viral overexpression of the complement inhibitor Crry at C3-bound synapses decreased the microglial engulfment of synapses and protected visual function.^97^ These therapeutic strategies, which have been generated for the treatment of neurodegenerative diseases, may also be applied to neurological complications derived from viral encephalitis.

## Summary points

Viral encephalitis has high mortality and morbidity, reflecting the lack of our understanding of the underlying mechanisms of CNS protection and damage.

Microglia, the innate immune cells that reside in the CNS parenchyma, deploy innate immune mechanisms to control virus spread shortly after CNS infection.

Microglia are nonredundant antigen-presenting cells in the CNS that regulate adaptive immune responses after infection.

Microglia are involved in CNS damage following the acute phase of viral encephalitis, which does not stop after virus elimination from the CNS.
